# Influence of Two Innovative Packaging Materials on Quality Parameters and Aromatic Fingerprint of Extra-Virgin Olive Oils

**DOI:** 10.3390/foods10050929

**Published:** 2021-04-23

**Authors:** Stefano Farris, Susanna Buratti, Simona Benedetti, Cesare Rovera, Ernestina Casiraghi, Cristina Alamprese

**Affiliations:** Department of Food, Environmental, and Nutritional Sciences (DeFENS), Università degli Studi di Milano, Via G. Celoria 2, 20133 Milan, Italy; stefano.farris@unimi.it (S.F.); simona.benedetti@unimi.it (S.B.); cesare.rovera@unimi.it (C.R.); ernestina.casiraghi@unimi.it (E.C.); cristina.alamprese@unimi.it (C.A.)

**Keywords:** electronic nose, accelerated shelf-life tests, transparent plastic material, metallized material, brown-amber glass, oxidation, stability, packaging, olive oil quality

## Abstract

The performance of two innovative packaging materials was investigated on two Sardinian extra-virgin olive oils (Nera di Gonnos and Bosana). In particular, a transparent plastic film loaded with a UV-blocker (packaging B) and a metallized material (packaging C) were compared each other and to brown-amber glass (packaging A). During accelerated shelf-life tests at 40 and 60 °C, the evolution of quality parameters (i.e., acidity, peroxide value, K_270_, and phenolic content) was monitored, together with the aromatic fingerprint evaluated by electronic nose. Packaging B resulted in the best-performing material in protecting oil from oxidation, due to its lower oxygen transmission rate (0.1 ± 0.02 cm^3^/m^2^ 24 h) compared to packaging C (0.23 ± 0.04 cm^3^/m^2^ 24 h). At the end of storage, phenolic reduction was on average 25% for packaging B and 58% for packaging C, and the aromatic fingerprint was better preserved in packaging B. In addition, other factors such as the sanitary status of the olives at harvesting and the storage temperature were demonstrated to have a significant role in the shelf life of packaged extra-virgin olive oil.

## 1. Introduction

The role of packaging throughout the food supply chain is of utmost importance since packaging contributes to preserving the food quality, maintaining the hygienic requisites thus preventing food-borne diseases, and allowing supply chain operations from the field to the consumer. Aside from these consolidated functions, modern and innovative packaging materials should be conceived to minimize their persistence into the environment, thus addressing the long-term crucial environmental issue of plastics disposal. At the same time, modern packaging is a key factor to address emerging challenges of sustainable food consumption, which involves the reduction of the environmental footprint of packed food [1]. From the environmental point of view, plastics are mistakenly perceived as the materials with the biggest environmental footprint mainly because they are almost exclusively seen under an end-of-life (EOL) perspective, with no consideration of material recyclability and impacts associated with the production and transport of the packaging materials [2,3].

However, plastic packaging materials, especially in the form of flexible configurations, provide environmental advantages and benefits over other materials, especially rigid configurations. For example, polyethylene terephthalate (PET) has demonstrated better environmental performance than traditional materials (e.g., aluminum and glass) in terms of consumption of natural resources and emissions [4]. In another work, it was demonstrated that bag-in-box and aseptic cartons had lower environmental impacts compared to single-use glass bottles for wine for all the impact categories considered by the authors, such as global warming potential, water consumption, and land use [5]. Besides, flexible packaging materials present transportation benefits since they are usually shipped either flat on a roll, thus allowing a dramatic reduction of trucks needed for transportation compared to rigid packaging [6].

Due to the high concentration of unsaturated fatty acids (oleic acid, linoleic acid, and linolenic acid), extra-virgin olive oil (EVOO) is subject to oxidation during storage even in the presence of abundant antioxidants (i.e., phenolic compounds and tocopherols). Oxidation is the main process affecting the quality of olive oil since some unstable compounds that can modify sensory and nutritional characteristics are produced. The level of EVOO oxidative degradation is strongly influenced not only by the chemical composition but also by the storage conditions. Packaging is therefore of great importance in preserving the quality of olive oil by protecting the product from oxygen and light.

Glass represents the first choice for EVOO because it is inherently impermeable to gases and vapors and it can be given light-filtering attributes, but, according to the above considerations, it may be of interest to evaluate packaging solutions for EVOO other than rigid glass. Pristouri et al. [7] compared the performance of glass bottles with plastic containers of the same volume (500 mL). They found that PET offered a moderately good performance, whereas polyethylene (PE) and polypropylene (PP) did not, mainly due to their low oxygen barrier properties. Gargouri et al. [8] evaluated the stability of Chemlali EVOO during storage with different packaging materials, i.e., clear and dark glass bottles, PE, and tin containers. They found that PE was inadequate to preserve EVOO from oxidation. Lolis et al. [9] investigated the effect of bag-in-box packaging material on the quality characteristics of EVOO using tinplate steel as the control. They demonstrated the best performance of the bag-in-box packaging materials, even when the EVOO samples were exposed to abuse temperatures (37 °C). In a more recent work, the same authors compared bag-in-box packaging material with dark-colored glass bottle [10]. They showed that samples packaged in bag-in-box material behaved in a similar way to those packaged in glass bottles.

In this work, we evaluated the performance of two innovative packaging materials on EVOO from two Sardinian olive cultivars: Nera di Gonnos and Bosana. In particular, a transparent plastic material loaded with a UV-blocker and a metallized material were compared to brown-amber glass bottles by monitoring the trends of oil quality indices (i.e., acidity, peroxide value, K_270_, and total phenolic content) during accelerated shelf-life tests conducted at two different temperatures (40 and 60 °C).

Moreover, a commercial electronic nose (e-nose) was used to follow the evolution of the aromatic fingerprint of Nera and Bosana oils during storage. E-nose is an instrument designed to mimic the human sense of smell, widely applied in determining the quality of foods. Compared with traditional analytical techniques, including gas chromatography, high-performance liquid chromatography, and spectroscopy, e-nose is relatively inexpensive and less time-consuming; compared with sensory evaluation, e-nose provides more objective and consistent measurements [11].

## 2. Materials and Methods

### 2.1. Packaging Materials

**Figure 1:**
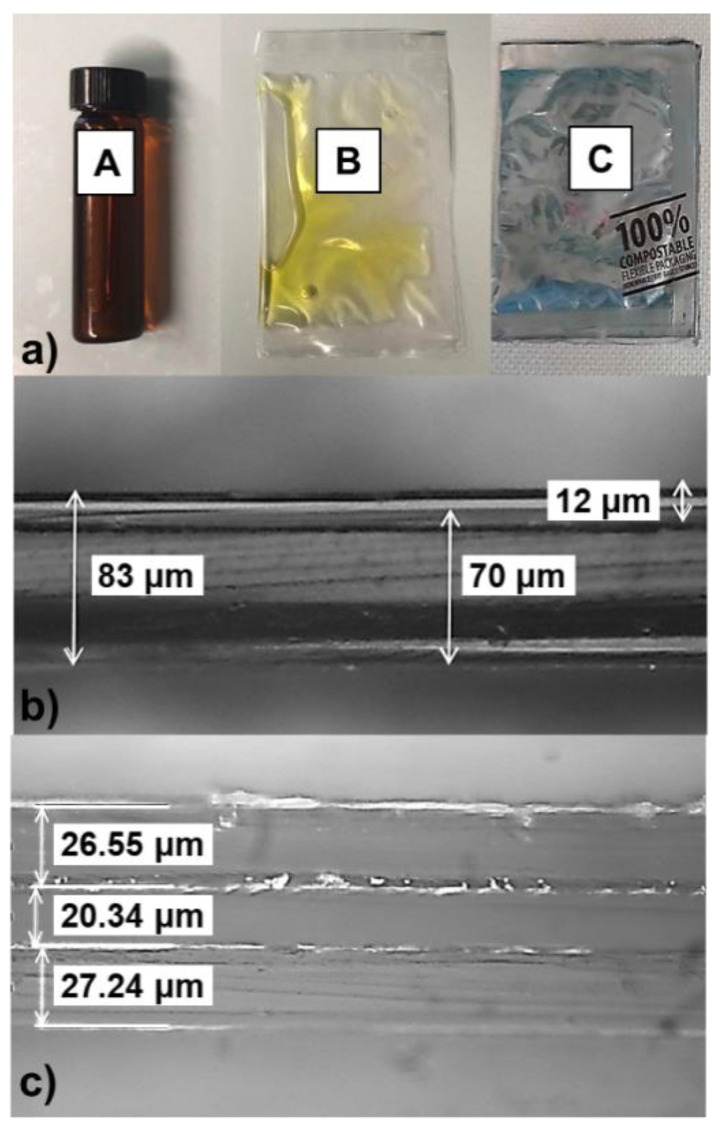
Packaging configurations used in the study: (**a**) glass vial (packaging A), transparent pouch (packaging B), and metallized pouch (packaging C); (**b**) optical microscope cross-sectional image (50 ×) of packaging B; (**c**) optical microscope cross-sectional image (50 ×) of packaging C.

Three different packaging materials were used in this study (Figure 1).

Brown-amber glass bottles (8 mL capacity) with a butyl/Teflon screw cap (Soffieria Vetro snc, Milano, Italy) were used as control and denoted as packaging A (Figure 1a). A transparent plastic film loaded with a UV-blocker (Cartastampa srl, Fornaci, Italy) was used as first testing material (coded as packaging B). In particular, it is a high-oxygen barrier film made of a 70 μm thick low-density polyethylene (LDPE) as the inner (in contact with oil) layer, coupled with a 12 μm high oxygen barrier-coated polyethylene terephthalate (PET) by means of a double-component polyurethane adhesive (Figure 1b). A second flexible material (coded as packaging C) was made of a metallized layer (20 μm) sandwiched between an external printable layer (25 μm) and an inner sealable layer (25 μm) (Figure 1c); according to the manufacturer (TIPA, Hod Hasharon, Israel), the final material is 100% compostable and up to 65% made of bio-based materials.

Pouches 11.5 × 7 cm were prepared using a thermal heat sealer Polikrimper TX/08 (Alipack, Pontecurone, Italy), provided by smooth bars at 140 °C for 0.5 s and 4.5 bar pressure.

### 2.2. Olive Oil Samples

Two Sardinian monovarietal EVOOs (Nera di Gonnos and Bosana cultivars) that differed mainly for natural antioxidant content were subjected to accelerated shelf-life tests (ASLT): both EVOOs were divided in 6 g aliquots, stored in the three different packaging materials, and kept in the dark at 40 ± 1 °C and 60 ± 1 °C up to 96 and 32 days, respectively. During storage, at scheduled times three aliquots, for each packaging of the two EVOOs were analyzed for quality parameters and e-nose aromatic profile.

### 2.3. Oxygen Barrier Properties of Packaging Films

Oxygen transmission rate (OTR, mL/m^2^ 24 h) was measured on a 50 cm^2^ surface sample using a PermeO_2_ permeabilimeter (PermTech srl, Pieve Fosciana, Italy) equipped with an electrochemical sensor, according to ASTM 3985, with a carrier flow (N_2_) of 10 mL/min at 23 °C and 0% relative humidity (RH) and at 1 atm pressure difference on the two sides of specimen. Three specimens were analyzed for each packaging materials.

### 2.4. Olive Oil Quality Parameters

The following quality parameters were considered:

- Acidity, indicative of the free fatty acid content and expressed as oleic acid (% oleic acid);
- Peroxide value (PV) corresponding to the amount of hydroperoxides (meq O_2_kg^−1^);
- Specific extinction at 270 nm (K_270_) providing a measurement of the secondary oxidation products.

All these analyses were performed in duplicate on each oil sample according to the methods reported in the European Regulation EEC no 2568/1991 and later amendments [12].

- Total phenolic content (TPC): oil samples were extracted with pure methanol as follows: 2 g oil was added to 5 mL methanol in a centrifuge tube, and the mixture was sonicated for 15 min. After sonication, the tube was centrifuged at 3500 rpm for 15 min at 15 °C, and the methanolic phase (extract) was separated; each sample was extracted in duplicate. TPC were determined by the Folin–Ciocalteu method [13], modified as follow: 0.5 mL of extract was added with 2.5 mL distilled water, 0.5 mL Folin–Ciocalteu reagent, and 2 mL Na_2_CO_3_ 10%, and the mixture was taken to 10 mL with distilled water. After 90 min rest in the dark, the mixture was filtered with 0.2 mm Whatman filter, and the absorbance was read at 750 nm (Spectrophotometer V-650, Jasco, Japan). Results were expressed as gallic acid equivalents (mg_GAE_ kg^−1^). Each extract was analyzed in duplicate.

### 2.5. Electronic Nose Analysis

Analyses were performed with the portable PEN3 e-nose (Airsense Analytics, Schwerin, Germany). The system is composed of a sampling apparatus, a sensor chamber containing the sensor array, and a pattern recognition software (Win Muster v.1.6) for data recording and processing. The sensor array consists of 10 metal oxide semiconductor (MOS) sensors: W1C (aromatic), W5S (broad range), W3C (aromatic), W6S (hydrogen), W5C (aromatic-aliphatic), W1S (broad-range), W1W (sulfur compounds), W2S (alcohols), W2W (sulfur compounds), and W3S (methane-aliphatic). The sensor response is expressed as resistivity (Ohm).

Two grams of oil samples were placed in 30 mL Pyrex^®^ vials fitted with a pierceable silicon/teflon disk in the cap. After 10 min at 40 °C ± 1 °C for the development of the headspace, the measurement started. The volatile compounds were pumped over the sensor surfaces for 60 s (injection time) at a flow rate of 300 mL min^−1^; the sensor signals were acquired at 50 s of sampling and statistically elaborated. After sample analysis, sensors were purged for 600 s with filtered air (purging time); then, prior to the next sample injection, the sensor baselines were re-established for 5 s. The sensor drift was estimate by using a standard solution of 0.2% ethanol included in each measurement cycle. The sensitivity of the instrument to various volatile compounds ranges from 0.1 and 5.0 ppm depending on their nature [14]. Each olive oil sample was evaluated in duplicate.

### 2.6. Data Analysis

Chemical data were analyzed by means of multifactor analysis of covariance (MANCOVA), considering the packaging material, EVOO cultivar, and storage temperature as main factors, and storage time as covariate. Two-way interaction effects were also evaluated. After checking the normal distribution of the responses, only TPC needed a squared transformation in order to fulfill the normality assumption. For significant factors, the Fisher’s least significant difference (LSD) procedure was applied for mean comparisons (*p* < 0.05). Data were processed by Statgraphics Centurion software (v. 18.1; Statgraphics Technologies Inc., The Plains, VA, USA).

E-nose data were analyzed by principal component analysis (PCA), an unsupervised technique used to pre-process and reduce the dimensionality of high-dimensional datasets while preserving the original structure and relationships inherent to the original dataset. PCA reduces the number of the original variables into unobservable variables (principal components) that are linear combinations of the original ones. The main purpose of PCA is the explanation of the variability of the original dataset with as few principal components as possible, thus allowing one to visualize the data structure and the relationships between objects (score plot) and how strongly each variable influences a principal component (loading plot) [15].

PCA can be performed in covariance or correlation matrix: if the variables studied are measured using the same scale, it is reasonable to use the covariance matrix to obtain the PCs; on the other hand, if the variables are measured in different scales, the correlation matrix must be applied as the original variables are all standardized to unit variance [16]. In this work, PCA was performed in covariance matrix since the scale is the same for all the e-nose sensors.

E-nose data were elaborated by Minitab 17 (v. 1.0, Minitab Inc., State College, PA, USA) software package.

## 3. Results and Discussion

### 3.1. Quality Parameters of Olive Oils Stored under Different Conditions

Accelerated shelf-life tests were carried out in order to compare the ability of the proposed packaging materials to preserve the quality of EVOO by preventing oxidation, which causes loss of nutritional value and defects in the sensory properties [17,18].

Since the experimental factors were the packaging material, the oil cultivar, and the storage temperature, a multifactor analysis of variance was performed on quality parameters, using the storage time as covariate, as it was correlated to all the responses. Thus, the real effect of each experimental factor was assessed, after adjusting the storage time effect. Table 1 shows the results obtained, in terms of significance of the main and interaction effects. As expected, storage time significantly affected all the parameters, covariating with them. All the considered experimental factors were also significant, with the exception of storage temperature for acidity.

**Figure 2:**
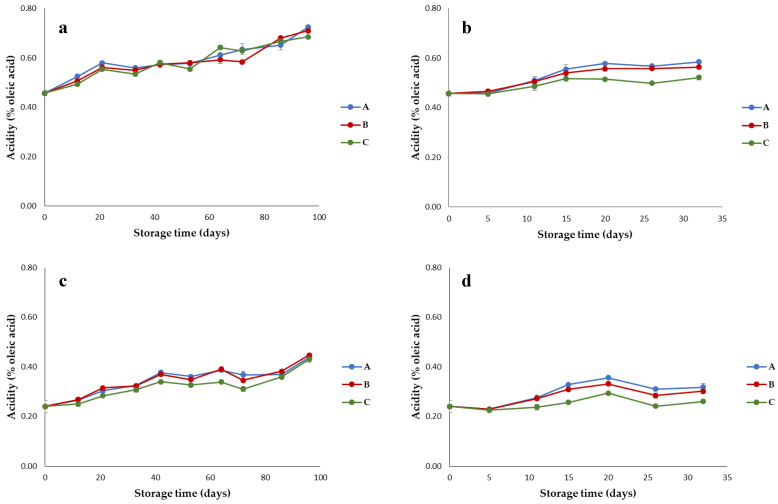
Trend of acidity in EVOOs stored in (A) brown-amber glass bottles, (B) transparent plastic material, and (C) metallized material; (**a**) Nera oil at 40 °C; (**b**) Nera oil at 60 °C; (**c**) Bosana oil at 40 °C; (**d**) Bosana oil at 60 °C. Error bars represent the standard deviation values (ranging from 0.001 to 0.024%).

Actually, a similar and progressive, though limited, acidity increase was observed at both 40 and 60 °C (Figure 2), as a consequence of the hydrolysis of triglycerides due to the action of lipases present in olives and produced by yeasts [19]. Nera oil showed acidity values significantly higher than that of Bosana oil (*p* < 0.001; Table 1), probably caused by a different sanitary status of the olives at harvesting. Indeed, excessive free fatty acids are associated with large, fully ripened, and fungus-infected drupes obtained from trees with low fruit loads. Even a small amount of such olives can spoil the oil quality [20]. However, different polyphenol content can also affected the acidity value since a higher phenolic concentration inhibits the activity of the lipase-producer yeasts [21]. At 40 °C, a similar increase of free fatty acid percentage was observed for the three packaging materials, whereas at 60 °C the highest acidity (about 0.58% and 0.32% for Nera and Bosana EVOOs, respectively) was evidenced for the oils stored in brown-amber glass bottles (packaging A) and in the transparent plastic material (packaging B). Indeed, the packaging × storage temperature effect was significant (*p* < 0.001), evidencing lower values of acidity at 60 °C for packaging C (metallized material) (Table 1). Anyhow, all the collected values did not exceed the limit of 0.8% set by the European Legislation [22].

**Figure 3:**
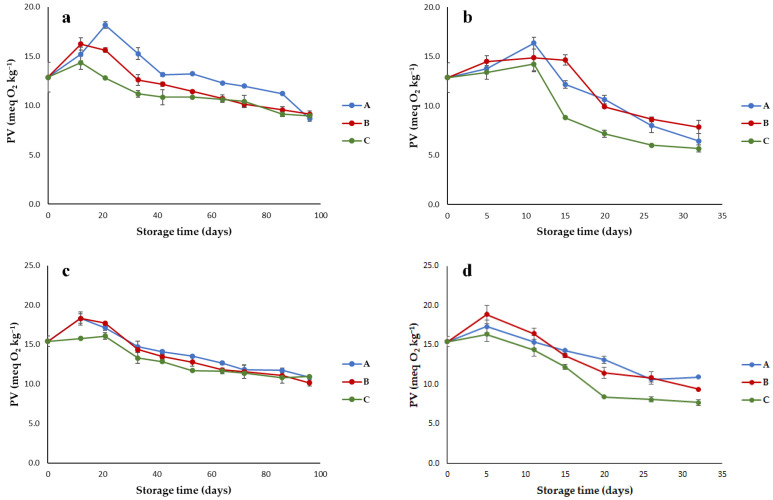
Trend of peroxide value (PV) in EVOOs stored in (A) brown-amber glass bottles, (B) transparent plastic material, and (C) metallized material; (**a**) Nera oil at 40 °C; (**b**) Nera oil at 60 °C; (**c**) Bosana oil at 40 °C; (**d**) Bosana oil at 60 °C. Error bars represent the standard deviation values (ranging from 0.01 to 1.51 meq O_2_kg^−1^).

Regarding the evolution of PV, after a first slight increase, a decrease was observed; thus, the legal limit of 20 meq O_2_kg^−1^ [22] was never reached (Figure 3). This trend can be explained by considering that PV decreases with the appearance of secondary oxidation products. During oxidation, the hydroperoxides can form and at the same time decompose; when the decomposition rate prevails, PV is lowered even before exceeding the legal limit if the temperature is high and the oxygen concentration low [23].

All the considered experimental factors significantly affected PV (*p* < 0.001), whereas the two-way interactions were all not significant (Table 1). The lowest values were observed for oils stored in the metallized material (C), especially when stored at 60 °C. This means that with this packaging the oil is less protected toward oxidation since the formation of secondary oxidation products is faster. A plausible explanation for this result is the different oxygen barrier performance of the three packaging materials. Indeed, the metallized material had a permeability to gas higher than that of packaging B and A. Brown-amber glass bottles are impermeable to oxygen and packaging B had an OTR of 0.1 ± 0.02 cm^3^/m^2^ 24 h, whereas OTR of packaging C was approximately double (0.23 ± 0.04 cm^3^/m^2^ 24 h). Moreover, the possible contact of the oil with the metallized side of the inner layer could have catalyzed the oxidation reactions.

As expected, due to the acceleration of oxidation reactions, storage at 60 °C caused a significantly higher decrease of PV.

The two EVOO cultivars showed significantly different PV (*p* < 0.001; Table 1), with the higher values in Bosana oil. For Nera oil, the initial PV was 12.9 meq O_2_kg^−1^; during storage at 40 °C, the maximum PV was reached at 21 days in the packaging A and 12 days in the packaging B and C; then, hydroperoxides readily decomposed to aldehydes, ketones, acids, esters, alcohols, and short-chain hydrocarbons [24], and PV gradually decreased to about 9.0 meq O_2_kg^−1^ at the end of storage (Figure 3a). A similar trend was observed for Nera oil stored at 60 °C (Figure 3b), with lower PV at the end of storage (5.7–7.8 meq O_2_kg^−1^) due to a faster degradation of peroxides to secondary oxidation products. The same PV evolution was observed for Bosana EVOO at both 40 (Figure 3c) and 60 °C (Figure 3d). Starting from an initial value of 15.4 meq O_2_kg^−1^, this parameter first increased and then decreased; in particular, at 60 °C, the hydroperoxide decomposition was prevalent just after 5 days of storage, and the final PV was between 7.7 and 10.9 meq O_2_kg^−1^. On average, at the end of storage Nera oil showed lower PV values than Bosana oil, probably due to a different polyphenol content affecting the protection toward oxidation phenomena.

**Figure 4:**
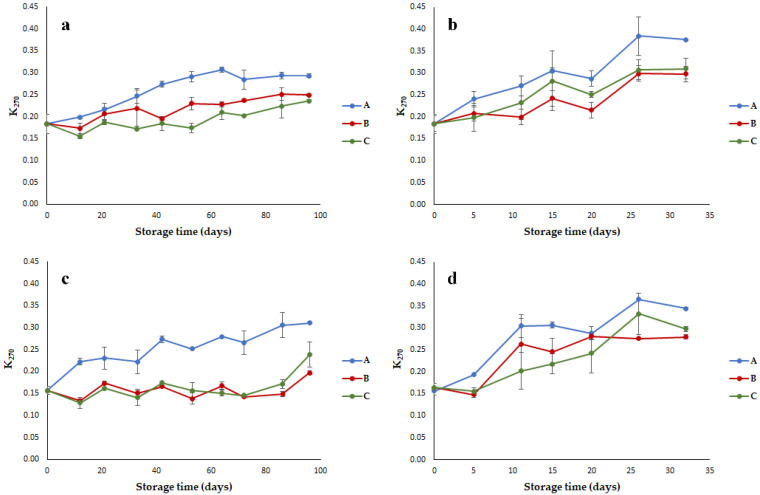
Trend of the spectrophotometric index K_270_ in EVOOs stored in (A) brown-amber glass bottles, (B) transparent plastic material, and (C) metallized material; (**a**) Nera oil at 40 °C; (**b**) Nera oil at 60 °C; (**c**) Bosana oil at 40 °C; (**d**) Bosana oil at 60 °C. Error bars represent the standard deviation values (ranging from 0.001 to 0.068).

K_270_ is known to be a good marker of oxidation secondary stage because it is related to conjugated trienes and carbonyl compounds [25]. In a recent work, Conte et al. [23] found that K_270_ is the best index for allowing one to predict EVOO shelf life when an accelerated test is applied. For both EVOO cultivars, this parameter significantly (*p* < 0.001) increased during storage, with significantly higher values in Bosana oil at 60 °C (Table 1; Figure 4), as a logical consequence of the higher initial oxidation state of Bosana samples and the acceleration of chemical reactions at higher temperatures. The packaging material had a significant effect on K_270_ (*p* < 0.001); the oils stored in the two innovative packaging materials (B and C) showed lower values than the oils stored in the brown-amber glass. In particular, the significantly lowest values were observed for the oils packaged in the metallized material stored at 60 °C; indeed, the interaction packaging × storage temperature had significant results (*p* < 0.01).

For Nera oil stored in the brown-amber glass bottles (packaging A), a significant increase of K_270_ was observed, and the legal limit of 0.22 [22] was exceeded after 12 days at 40 °C and 11 days at 60 °C. For packaging B and C, the extinction at 270 nm increased considerably only at the end of storage at 40 °C, whereas at 60 °C, the legal limit was exceeded after 11 days for packaging B and 15 days for packaging C (Figure 4a,b). Similar results were obtained for Bosana oil (Figure 4c,d), with the samples packaged in brown-amber glass bottles characterized by a higher index of secondary oxidation products and exceeding the legal limit for K_270_ after 21 and 5 days of storage at 40 and 60 °C, respectively. This result could seem in contrast with the well-known protection ability of glass toward oil oxidation phenomena. However, in this case, the higher K_270_ values could be related to the higher retention capacity of glass toward low-molecular-weight compounds with respect to the tested innovative packaging materials.

**Figure 5:**
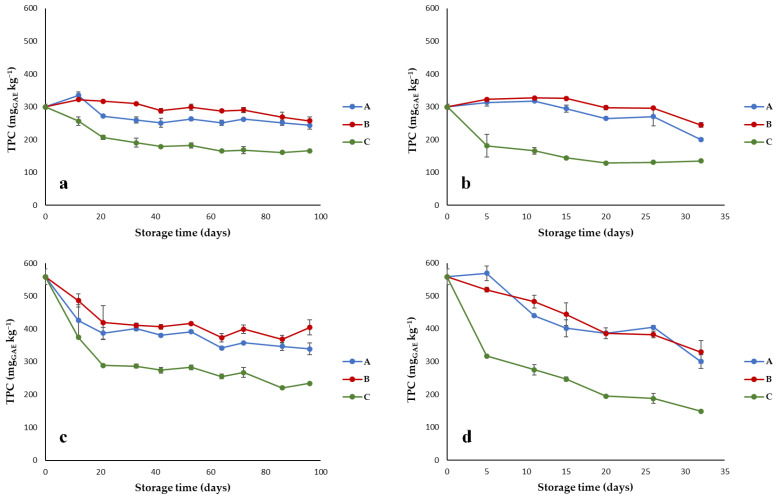
Trend of total phenolic content (TPC) in EVOOs stored in (A) brown-amber glass bottles, (B) transparent plastic material, and (C) metallized material; (**a**) Nera oil at 40 °C; (**b**) Nera oil at 60 °C; (**c**) Bosana oil at 40 °C; (**d**) Bosana oil at 60 °C. Error bars represent the standard deviation values (ranging from 0.1 to 47.6 mg_GAE_ kg^−1^).

Phenolic compounds are naturally present in olive oils, and they are responsible for oil stability during storage [26]. Figure 5 shows the evolution of TPC in Nera and Bosana oil as affected by packaging material and storage temperature.

Nera and Bosana oils were characterized by significantly different values of TPC, and the cultivar was indeed a significant factor (*p* < 0.001; Table 1) affecting this quality parameter during storage. Nera cultivar had a medium/low content of phenolics (300 mg_GAE_ kg^−1^), whereas Bosana was characterized by high polyphenol concentration (558 mg_GAE_ kg^−1^). During storage at both temperatures, a progressive decrease in TPC was observed (Figure 5), with a significantly (*p* < 0.001; Table 1) different trend for the tested packaging materials. In particular, the metallized material (packaging C) had a detrimental effect on phenolics, causing a higher TPC reduction, especially at 60 °C. In fact, the interaction packaging material x storage temperature was significant (*p* < 0.001). The average TPC reduction for the two EVOOs in packaging C at the end of storage was about 52% at 40 °C and 64% at 60 °C. The best-performing material in protecting oil phenolic content was the transparent plastic packaging (B), followed by the brown-amber glass (packaging A); this result can be again ascribed to the better oxygen barrier performance of the transparent pouches. During storage at 40 and 60 °C, TPC reduction was on average 21% and 30% for packaging B and 29% and 40% for packaging A. These results are in agreement with previous works showing that, during storage, phenolic compounds undergo quantitative modification due to oxidation and the temperature as well as the packaging material can have a notable influence on phenolic degradation [8,10].

### 3.2. E-Nose Aromatic Profile of Olive Oils Stored under Different Conditions

The e-nose is an instrument composed by non-selective or semi-selective sensors interacting with aromatic compounds to produce electronic signals. In the analysis of olive oil, e-nose has been successfully used in the determination of the geographical origin, in the detection of adulteration, and in the prediction of shelf-life [27].

In this work, a portable e-nose, with ten different MOS sensors, was applied in order to evaluate the effects of the three packaging materials on the evolution of the aromatic fingerprint of Nera and Bosana oil during storage, and the collected data were elaborated by PCA.

**Figure 6:**
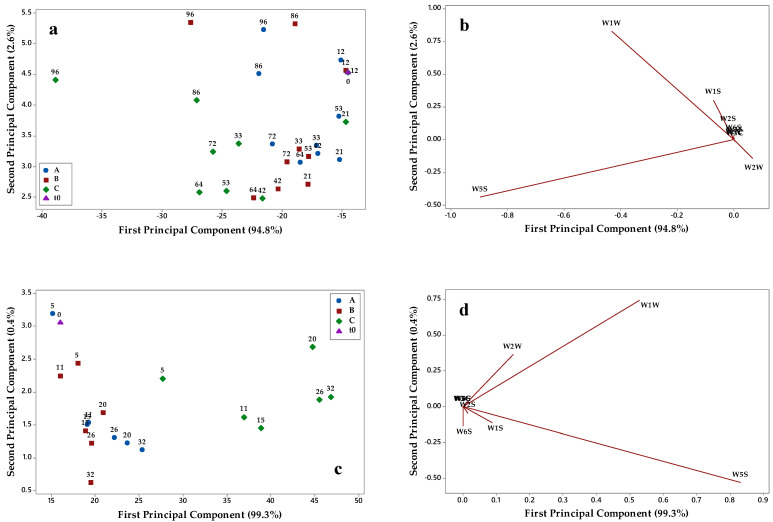
Results of principal component analysis of e-nose data of Nera oil stored in (A) brown-amber glass bottles, (B) transparent plastic material, and (C) metallized material: (**a**) score plot of samples stored at 40 °C; (**b**) loading plot for samples stored at 40 °C; (**c**) score plot of samples stored at 60 °C; (**d**) loading plot for samples stored at 60 °C.

**Figure 7:**
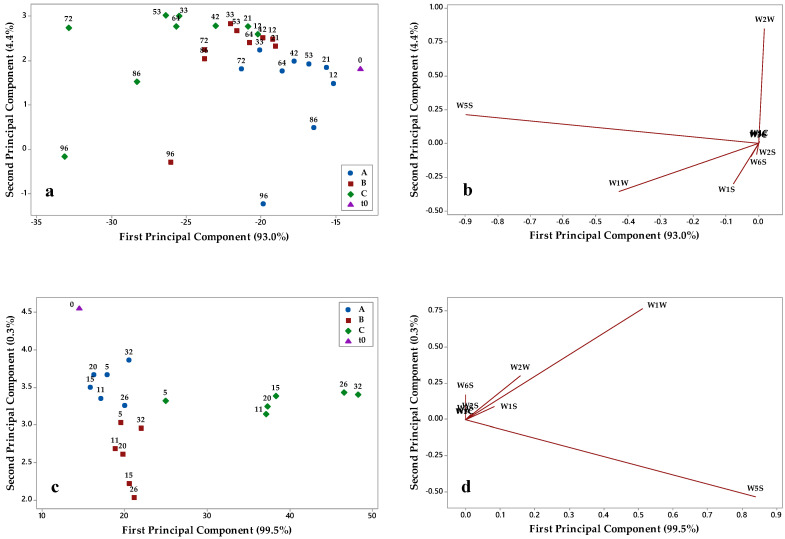
Results of principal component analysis of e-nose data of Bosana oil stored in (A) brown-amber glass bottles, (B) transparent plastic material, and (C) metallized material: (**a**) score plot of samples stored at 40 °C; (**b**) loading plot for samples stored at 40 °C; (**c**) score plot of samples stored at 60 °C; (**d**) loading plot for samples stored at 60 °C.

In order to evaluate the effects of the three packaging materials on the evolution of the aromatic fingerprint of Nera and Bosana oil during storage, a commercial e-nose was applied, and collected data were elaborated by PCA. Figure 6 and Figure 7 show the PCA score plots and loading plots of the two oils in the plane defined by the first two principal component (PC1 and PC2) explaining almost all the variance.

By examining the PCA score plots of Nera oil (Figure 6a,c), it can be seen that, at both temperatures, the sample distribution on PC1 and PC2 followed the storage time and was affected by the packaging materials. At 40 °C, samples stored in the packaging A and B were mainly located to the right of the plot, and their aromatic profile was similar to that of fresh oil (t0) up to about 64 days of storage. For longer times (i.e., from 72 to 96 days), samples were discriminated on PC2 and located in the upper part of the score plot. The evolution trend of the oil stored in packaging C was more significant; at both temperatures, samples were distributed on PC1 according to the storage time and the aromatic profile of the oil evolved rapidly after 21 days at 40 °C and 5 days at 60 °C. Similar considerations can be drawn from Bosana score plots (Figure 7a,c); at both 40 and 60 °C, the oil samples stored in packaging C were distributed on PC1 according to the storage time, and a rapid evolution of the aromatic profile was noticeable. The oil samples stored in packaging A and B were characterized by a less modified aromatic fingerprint and by a similar evolution trend on PC1 and PC2.

The e-nose findings were quite consistent with the phenolic degradation during storage (Figure 5), in agreement with previous works reporting the influence of polyphenolic compounds on the aroma of olive oil [28,29].

The loading plots of Nera (Figure 6b,d) and Bosana (Figure 7b,d) oils allowed one to relate e-nose sensors with the evolution trends in the score plots. W5S and W1W sensors were relevant on PC1, while W1S and W2W sensors discriminated samples on PC2. The W5S sensor was found to be the most relevant in the discriminating oil samples based on their storage conditions, and the same occurred in other works monitoring the evolution of the aromatic profile of vegetables during shelf life [30,31].

In a recent work, Xu et al. [14] evaluated the performance of PEN 3 e-nose to discriminate oils based on their oxidation rate. In agreement with our findings W5S, W1S, W1W, and W2W sensors showed different response signals to volatile compounds of oxidized oils compared to non-oxidized oils, thus demonstrating that this device provides a rapid and accurate method for characterizing the evolution aromatic profile of the oil during oxidation.

## 4. Conclusions

In spite of the large use of glass as main material for packaging and distribution of extra-virgin olive oil, increasing awareness of sustainability imposes a careful attention toward the selection of packaging materials with less overall environmental impact. According to this perspective, considering new materials other than glass bottles seems to be a trend that will not relent in the years ahead, especially as far as emerging markets are concerned. In this work, we have demonstrated that a flexible packaging material with outstanding oxygen barrier performance can be effectively used for extra-virgin olive oil, outperforming conventional glass as indicated by the most relevant quality parameters. More specifically, the oxygen barrier performance seemed to play the most important role in preserving the overall quality of EVOO, especially at high temperatures (e.g., 60 °C). In this regard, the oxidation of the EVOO was less pronounced when the packaging with the lowest OTR was used (packaging B). In addition, other factors such as the sanitary status of the olives at harvesting and the storage temperature have been demonstrated to have a significant role in the shelf life of packaged EVOO. Findings arising from this work can be profitably used for the design of new packaging configurations as sustainable alternatives to glass.

## Figures and Tables

**Table 1:** Results of MANCOVA (F-ratio and significance level) for oil quality parameters.

Source	Acidity	PV	K270	TPC (Squared)
Covariate				
Storage time	476.41 ***	206.32 ***	93.88 ***	129.81 ***
Main effects				
Packaging	10.13 ***	16.47 ***	51.02 ***	354.02 ***
Oil cultivar	4000.51 ***	36.97 ***	15.72 ***	486.69 ***
Storage temperature	2.91 n.s.	124.73 ***	176.04 ***	60.93 ***
Interactions				
Packaging × Cultivar	8.57 ***	0.14 n.s.	1.68 n.s.	2.53 n.s.
Packaging × Temperature	10.11 ***	2.55 n.s.	5.15 **	22.34 ***
Cultivar × Temperature	4.77 *	1.79 n.s.	3.06 n.s.	0.74 n.s.

PV, peroxide value; TPC, total phenolic content; n.s., not significant; *, *p* < 0.05; **, *p* < 0.01; ***, *p* < 0.001.

## Data Availability

Not applicable.
